# 25-Hydroxycholesterol Inhibition of Lassa Virus Infection through Aberrant GP1 Glycosylation

**DOI:** 10.1128/mBio.01808-16

**Published:** 2016-12-20

**Authors:** Punya Shrivastava-Ranjan, Éric Bergeron, Ayan K. Chakrabarti, César G. Albariño, Mike Flint, Stuart T. Nichol, Christina F. Spiropoulou

**Affiliations:** Viral Special Pathogens Branch, Division of High Consequence Pathogens and Pathology, Centers for Disease Control and Prevention, Atlanta, Georgia, USA

## Abstract

Lassa virus (LASV) infection is a major public health concern due to high fatality rates and limited effective treatment. The interferon-stimulated gene *cholesterol 25-hydroxylase* (*CH25H*) encodes an enzyme that catalyzes the production of 25-hydroxycholesterol (25HC). 25HC is involved in regulating cholesterol biosynthesis and has recently been identified as a potent antiviral targeting enveloped virus entry. Here, we show a previously unrecognized role of CH25H in inhibiting LASV glycoprotein glycosylation and the production of infectious virus. Overexpression of CH25H or treatment with 25HC decreased LASV G1 glycoprotein *N*-glycan maturation and reduced the production of infectious LASV. Depletion of endogenous CH25H using small interfering RNA (siRNA) enhanced the levels of fully glycosylated G1 and increased infectious LASV production. Finally, LASV particles produced from 25HC-treated cells were found to be less infectious, to incorporate aberrantly glycosylated GP1 species, and to be defective in binding alpha-dystroglycan, an attachment and entry receptor. Our findings identify a novel role for CH25H in controlling LASV propagation and indicate that manipulation of the expression of CH25H or the administration of 25HC may be a useful anti-LASV therapy.

## IMPORTANCE

Lassa fever is an acute viral hemorrhagic fever in humans caused by Lassa virus (LASV). No vaccine for LASV is currently available. Treatment is limited to the administration of ribavirin, which is only effective when given early in the course of illness. *Cholesterol 25-hydroxylase* (*CH25H*) is a recently identified interferon-stimulated gene (ISG); it encodes an enzyme that catalyzes the production of 25-hydroxycholesterol (25HC), which inhibits several viruses. Here, we identify a novel antiviral mechanism of 25HC that is dependent on inhibiting the glycosylation of Lassa virus (LASV) glycoprotein and reducing the infectivity of LASV as a means of suppressing viral replication. Since N-linked glycosylation is a critical feature of other enveloped-virus glycoproteins, 25HC may be a broad inhibitor of virus infectivity.

## INTRODUCTION

*Arenaviridae* is a family of enveloped, negative-strand RNA viruses that can cause severe disease in humans; it includes Lassa virus (LASV), lymphocytic choriomeningitis virus (LCMV), Lujo virus, Junin virus, and Machupo virus. LASV, the most prevalent cause of arenavirus infection, is endemic to West Africa and causes an estimated 300,000 infections annually, with case fatality rates as high as 20% (1, 2). LASV is classified as a biosafety level 4 (BSL-4) and NIAID biodefense category A agent (1). Patients infected with LASV have initial nonspecific symptoms similar to those of Ebola virus infection, including fever, headaches, muscle pain, weakness, fatigue, and vomiting. In severe cases, LASV disease can be associated with a bleeding diathesis; death occurs due to multiorgan failure (2, 3). No vaccine for LASV is currently approved, and ribavirin, the sole available drug, is only effective if administered early in infection (2, 4). The death of a patient with Lassa fever in the United States in 2015 (5) demonstrates that even excellent intensive care cannot always prevent a fatal outcome and emphasizes the need for developing effective vaccines and novel therapies.

The LASV genome consists of 2 single-stranded ambisense RNA segments, one large (L; 7.2 kb) and one small (S; 3.4 kb). L encodes the viral polymerase and the zinc-binding protein (Z or matrix protein). S encodes the nucleoprotein (NP) and glycoprotein precursor (GPC). The LASV GPC, synthesized as a single protein, is cleaved posttranslationally into GP1 and GP2 by the cellular enzyme site 1 protease SKI-1/S1P (6, 7). These subunits oligomerize, forming spikes on the surface of the virion. GP1 is responsible for virus attachment to host cell receptors, while GP2 mediates fusion of the viral and endosomal membranes (8, 9). GPC must be glycosylated for correct folding in order to be cleaved by SKI-1/S1P (10). In addition, glycosylation of GP1/GP2 is needed for their transport to sites of viral budding, as well as for LASV antigenicity and infectivity (6, 11, 12). Hence, the processes of LASV GPC cleavage, glycosylation, and maturation represent targets for potential antiviral strategies.

*Cholesterol 25-hydroxylase* (*CH25H*) is a newly identified, conserved interferon-stimulated gene (ISG) that encodes an enzyme that catalyzes the oxidation of cholesterol into 25-hydroxycholesterol (25HC) (13). The antiviral effects of CH25H have been predominantly attributed to its enzymatic activity through the production of 25HC, which has been shown to block infection by several enveloped viruses (14–17). The antiviral function of 25HC involves multiple mechanisms, including blocking fusion of the viral envelope with host membranes (14), inhibiting viral replication (14, 15), and inhibiting the formation of viral replication complexes on intracellular membranes (17). 25HC was also reported to inhibit a postentry step of viral infection by repressing the activation of SREBP (16), a transcription factor required for lipid and cholesterol biosynthesis. More recently, 25HC has also been shown to block the replication of nonenveloped viruses (18). These findings demonstrate that 25HC has broad-spectrum antiviral effects. Understanding the mechanisms by which 25HC exerts antiviral activity may help to identify strategies and targets for potential intervention.

Here, we report that 25HC restricted LASV infection by inducing the biosynthesis of an aberrantly glycosylated form of GP1, which was efficiently incorporated into LASV particles. Incorporation of the aberrantly glycosylated GP1 was accompanied by reduced LASV infectivity and binding to alpha-dystroglycan (alpha-DG), a cell surface protein involved in LASV attachment and endocytosis (19, 20). Our findings suggest that 25HC can restrict virus infection by a previously unrecognized mechanism linking improper glycosylation and binding of the viral glycoprotein to a cellular receptor.

## RESULTS

### 25HC impairs LASV production.

**FIG 1:**
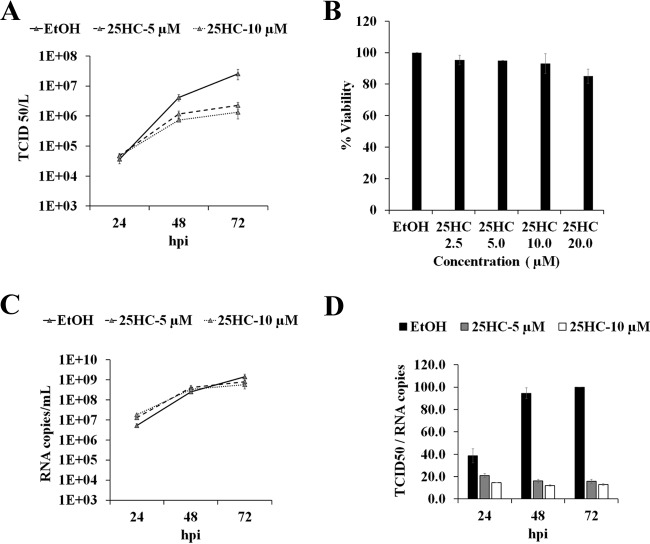
25-Hydroxycholesterol inhibits infectious Lassa virus production. (A) Culture supernatants of cells infected with Lassa virus (LASV; MOI of 0.1) pretreated with ethanol (EtOH; vehicle control) or indicated concentrations of 25-hydroxycholesterol (25HC) were harvested at different times postinfection (hours postinfection [hpi]), and viral titers were quantified by 50% tissue culture infective dose (TCID_50_) assay. (B) Cell viabilities (%) of 25HC-treated and mock-infected cells were determined after 72 h of treatment. Values were normalized to those of ethanol controls. (C) RNA was extracted from supernatants of cells infected as described for panel A. Absolute quantification of viral RNA copy numbers was done using a standard curve with known viral titers. Mean RNA copy numbers are shown, with error bars indicating standard deviations calculated from the results of 3 independent experiments. (D) Ratios were calculated using TCID_50_/ml values from the experiment whose results are shown in panel A divided by the extracellular viral RNA copy numbers from the experiment whose results are shown in panel B. Mean specific infectivity was calculated as the percentage of the mean value for ethanol-treated samples at 72 hpi, with error bars indicating standard deviations calculated from the results of 3 independent experiments.

We first tested the ability of 25HC to reduce the production of infectious LASV. Huh7 cells were treated for 1 h with either ethanol (vehicle control) or 5 or 10 μM of 25HC and then infected with LASV at a multiplicity of infection (MOI) of 0.1. The LASV titers were reduced by both concentrations of 25HC at 48 and 72 h postinfection (hpi). At 5 μM, 25HC inhibited the production of infectious LASV titers by >1.1 log, and 10 μM of 25HC inhibited LASV titers by 1.4 log (Fig. 1A). These decreases in viral titers were not due to cellular toxicity, as no significant cytotoxic effects were measured over the same time period (Fig. 1B).

### LASV infectivity is reduced by 25HC treatment.

Next, we used quantitative reverse transcription-PCR (qRT-PCR) to determine LASV RNA copy numbers in supernatants of LASV-infected cells. In contrast to LASV infectious titers (Fig. 1A), the extracellular LASV RNA levels from control and 25HC-treated samples increased to 1 × 10^9^ copies per ml at 72 hpi, and 25HC treatment did not affect viral RNA release (Fig. 1C). To highlight the differences between LASV titers and viral RNA copy numbers due to 25HC treatment, we calculated the ratio of infectious LASV (50% tissue culture infective dose [TCID_50_]/ml) to viral RNA copy number (Fig. 1D). In 25HC-treated cells, the ratio was reduced to ~15% of the values for controls at 48 and 72 hpi, suggesting that the loss in LASV infectivity was most notable after 24 hpi.

Previous reports suggest that 25HC blocks cell entry of enveloped viruses (14). To assess whether the impaired LASV infectivity was due to inhibited LASV entry, we measured the levels of cell-associated LASV S genome at an early time point after infection. Huh7 cells pretreated with 25HC were infected with LASV at an MOI of 1. S segment RNA levels were measured by qRT-PCR and normalized to the level of glyceraldehyde-3-phosphate dehydrogenase (GAPDH) mRNA. As shown by the results in Fig. S1 in the supplemental material, the S genome levels did not differ significantly among the samples, indicating that 25HC did not affect the levels of internalized LASV genome. On the other hand, treatment with the positive-control BIBX 1382, previously shown to inhibit LASV glycoprotein-dependent entry (21), significantly reduced the S genome levels. These results are consistent with 25HC not affecting the initial stages of LASV entry.

### 25HC alters the electrophoretic mobility of glycosylated GP1.

**FIG 2:**
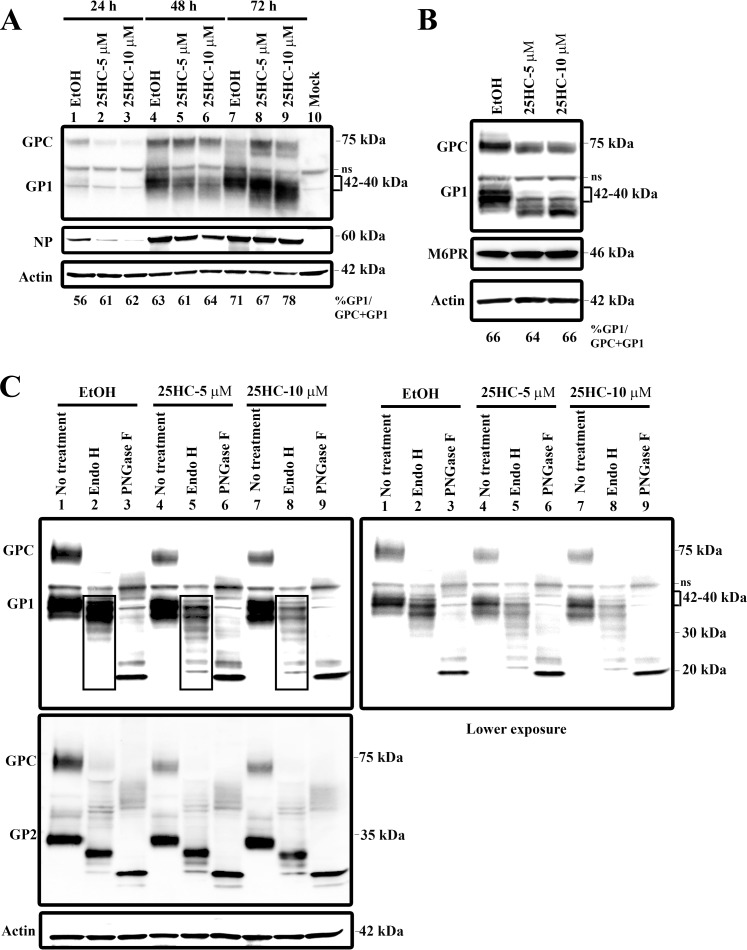
25HC treatment affects LASV GP1 glycosylation. (A) Huh7 cells were infected with LASV and treated with ethanol or 25HC as described in the legend to Fig. 1A. Cell lysates were harvested at different times after infection, and GP1 and actin expression levels were determined by Western blotting. ns, nonspecific binding. (B) Huh7 cells were transfected with a plasmid expressing LASV GPC and treated with 25HC. The levels of GP1 and actin expression in cell lysates were then analyzed by Western blotting. To determine the extent of GPC cleavage, blot images were subjected to densitometry analysis. Percentage of GP1 was determined by dividing the GP1 signal by the total amount of glycoprotein recognized by GP1 MAb (GP1/GPC + GP1). (C) Cell lysates prepared from 25HC treated and LASV-GPC transfected cells as described for panel B were either left untreated or were digested with Endo-H or PNGase. The levels of GP1, GP2, and actin expression were then visualized by Western blotting.

To understand the mechanism by which 25HC reduces LASV infectivity, we examined the viral proteins in the lysates of LASV-infected cells. Western blot analysis using a monoclonal antibody (MAb) recognizing the GP1 region detected GPC migrating at ~75 kDa and the cleavage product GP1 as a diffuse 40- to 42-kDa band in lysates of untreated cells (Fig. 2A). At 48 h, treatment with 5 and 10 μM 25HC decreased the levels of the 42-kDa species but not the 75-kDa species. At 72 hpi, the intensity of the 42-kDa band shifted below ~40 kDa. 25HC treatment also changed the GPC levels (Fig. 2A, lane 7 versus lane 9), but the ratio of GP1 cleavage efficiency [%GP1/(GP1 + GPC)] was unaffected. 25HC treatment caused decreased LASV NP levels at 24 and 48 hpi, but the NP levels were similar to those in the vehicle control at 72 hpi. Thus, 25HC increased GP1 mobility on SDS-PAGE gels and reduced infectious LASV production (Fig. 2A and 1A).

### 25HC inhibits LASV GP1 glycosylation in LASV GPC-transfected cells.

Cleavage of LASV GPC by SKI-1/S1P is essential for the production of infectious virus. The reduced LASV titers in 25HC-treated cells (Fig. 1A) could be due to reduced cleavage or reduced glycosylation of LASV GPC. Thus, we next looked at the expression of GPC and GP1 in Huh7 cells transfected with LASV GPC and treated with 25HC or ethanol vehicle control. Cells were treated with 25HC, and cell lysates were analyzed by Western blotting with antibodies for GP1 or actin. GPC-transfected and ethanol-treated cells showed GPC and GP1 electrophoretic mobilities similar to those of the LASV-infected cells (compare Fig. 2B and A), with the precursor protein migrating at ~75 kDa and the majority of GP1 detected at ~40 to 42 kDa. After 24 h of treatment, 25HC did not have a significant effect on GP1 glycosylation in GPC-transfected cells (see Fig. S2 in the supplemental material). However, after 48 h, similar to the results for LASV-infected cells, 5 µM or 10 µM of 25HC resulted in a distinct shift in GP1 mobility on SDS-PAGE gels, from ~40 to 42 kDa to below ~40 kDa. The unprocessed GPC band shifted also, from ~75 kDa to ~73 kDa, after 25HC treatment. Although the overall levels of GPC and GP1 appeared slightly reduced upon 25HC treatment, 25HC did not affect GPC cleavage efficiency, as demonstrated by quantifying GP1 and GPC levels in cell lysates (Fig. 2B). No effect on the level or the migration of the endogenous protein mannose 6-phosphate receptor (M6PR), which contains 5 potential N-linked glycosylation sites (22), was detected by 25HC treatment. Thus, 25HC specifically increased GP1 mobility on SDS-PAGE gels but did not affect GPC cleavage efficiency in the absence of other viral proteins.

To test whether the change in GP1 mobility on SDS-PAGE gels was due to changes in glycosylation, the lysates of cells transfected with LASV GPC were treated with endoglycosidase H (Endo-H) or peptide-*N*-glycosidase F (PNGase F). Treatment with PNGase F, which removes all N-linked glycans, caused the majority of GP1 to migrate at between ~20 and ~24 kDa (instead of ~40 to 42); this pattern was observed regardless of whether 25HC was used (Fig. 2C, lanes 3, 6, and 9). Without 25HC, Endo-H digestion, which removes high-mannose species while leaving the most complex and hybrid glycans intact, resulted in a shift of intensity of the ~40- to 42-kDa GP1 band to just below ~40 kDa in the lysates of control cells (Fig. 2C, lane 1 versus lane 2). In contrast, when 25HC-treated lysates were digested with Endo-H, the intensity of the GP1 species migrating below ~40 kDa was decreased, and multiple additional GP1 bands migrating from ~40 kDa to ~22 kDa became apparent (Fig. 2C, lanes 2, 5, and 8, top). Together, this indicated that the GP1 species below ~40 kDa are more sensitive to Endo-H digestion in 25HC-treated lysates than in lysates of control cells; this increase in sensitivity suggests that the maturation of GP1 *N*-glycans was impaired by 25HC. In contrast, GP2 sensitivity to Endo-H digestion was minimally affected in the presence and absence of 25HC (Fig. 2C lanes 2, 5, and 8, bottom). Taken together, these results indicate that 25HC treatment leads to the production of aberrantly glycosylated forms of GP1 that contain more immature *N*-glycans.

### Overexpression of CH25H alters GP1 electrophoretic mobility and LASV production.

**FIG 3:**
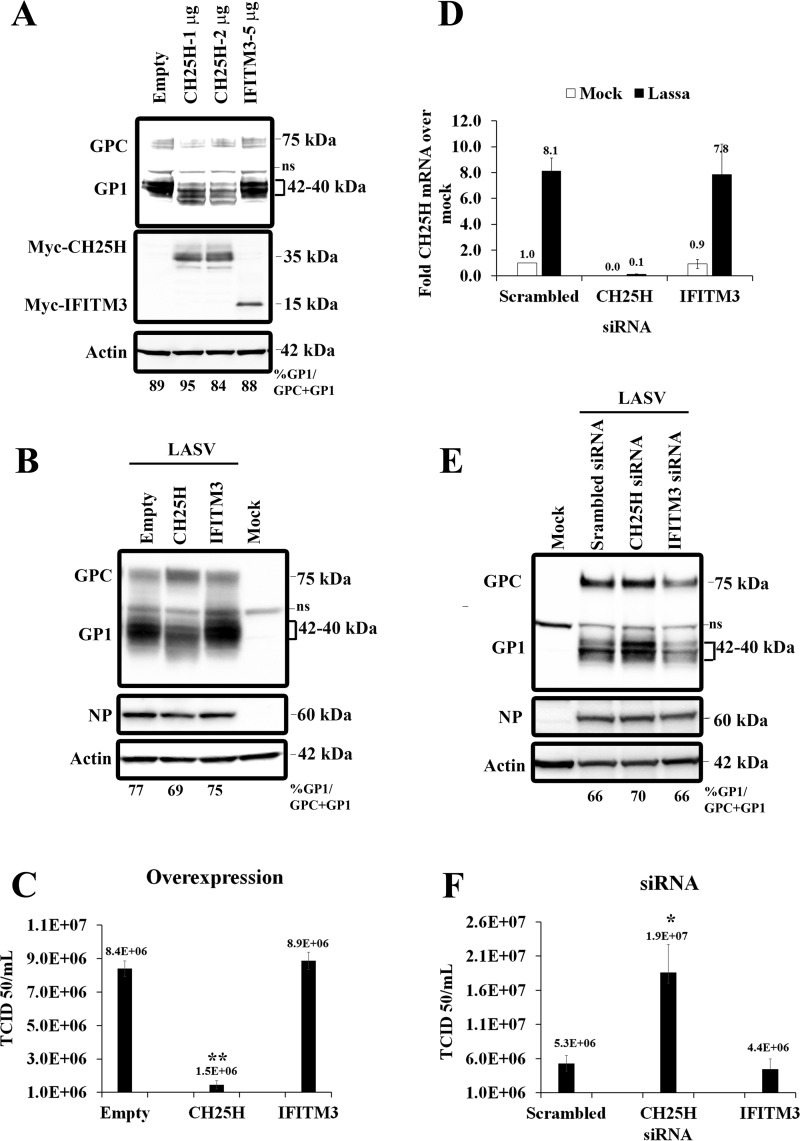
Effects of CH25H overexpression and knockdown on LASV infectivity and GP1. (A) Huh7 cells were transfected with empty vector or plasmid expressing Myc-tagged cholesterol 25-hydroxylase (CH25H) or Myc-tagged IFITM3. After 24 h, the cells were transfected with LASV GPC. Transfected cells were harvested 48 h later, and GP1 was detected by Western blotting. The membranes were stripped and reprobed, first with anti-Myc antibody and then with antiactin antibody. ns, nonspecific binding. (B) Huh7 cells transfected with CH25H or IFITM3 were infected with LASV at an MOI of 0.1 24 h posttransfection. Cell lysates and supernatants were collected at 72 hpi. Western blotting was used to detect GP1, NP, and actin. (C) Huh7 cells were transfected with plasmid expressing CH25H or IFITM3 and infected with LASV. Cell supernatants were collected at indicated times after infection, and LASV titers were determined by TCID_50_ assays in Vero-E6 cells. (D) Huh7 cells were transfected with the indicated siRNAs and then either mock infected or infected with LASV at an MOI of 1.0 in medium containing 5% lipoprotein-deficient serum. After 24 h, lysates were prepared, and CH25H expression was analyzed by qRT-PCR. CH25H levels were normalized to levels of GAPDH in each sample. (E) Huh7 cells were transfected with indicated siRNAs and infected with LASV. After 24 h, lysates were collected, and Western blotting was used to detect GP1, NP, and actin. (F) Huh7 cells were transfected and infected with LASV as described for panel E. After 24 h, supernatants of infected cells were collected, and LASV titers were determined by TCID_50_ assays in Vero-E6 cells. *, *P* < 0.05; **, *P* < 0.005.

25HC is generated by the enzyme CH25H. To confirm a role for CH25H in GPC glycosylation, we overexpressed this enzyme in Huh7 cells. Huh7 cells transfected with Myc-tagged CH25H or with empty plasmid were transfected with LASV GPC, and GP1 expression was analyzed by Western blotting. As a negative control, we used Myc-tagged IFITM3, another ISG that can block virus entry by disrupting intracellular cholesterol homeostasis (23, 24). The expression of CH25H and IFITM3 was confirmed by Western blotting (Fig. 3A). As seen with 25HC treatment, CH25H transfection altered the migration of GP1 to below 40 kDa (Fig. 3A). In contrast, no shift in GP1 band intensity was observed in cells transfected with either empty plasmid or IFITM3.

To investigate whether the expression of CH25H also affected GP1 glycosylation during LASV infection, as it does in GPC-transfected cells, we overexpressed CH25H in Huh7 cells and infected these cells with LASV. The expression of CH25H, but not of IFITM3, shifted the GP1 band at 72 hpi (Fig. 3B), similar to 25HC treatment (Fig. 2A). Furthermore, cells overexpressing CH25H produced 5 times less LASV than infected cells that received the empty vector (Fig. 3C). In comparison, IFITM3 overexpression did not affect LASV growth (Fig. 3C). Thus, CH25H overexpression altered LASV GP1 glycosylation and decreased LASV production. Taken together, the similar effects of CH25H expression and 25HC treatment suggest that 25HC synthesized by CH25H is responsible for the effects of CH25H transfection.

### Knocking down CH25H expression increases LASV production.

To evaluate the role of endogenous CH25H in LASV GP1 glycosylation and the reduction of LASV titers, we silenced CH25H mRNA expression under sterol-depleted conditions. Sterol depletion was used to increase CH25H expression to help detect changes in GP1 glycosylation and LASV titers. In LASV-infected cells, small interfering RNA (siRNA) targeting CH25H reduced its expression by more than 85% (Fig. 3D), and no change in CH25H levels was observed in control cells treated with siRNA targeting IFITM3 or scrambled siRNA. Cells treated with CH25H siRNA showed increased intensity in the GP1 band at 42 kDa, consistent with more complex *N*-glycan content in GP1 (Fig. 3E). Increased intensity of the 42-kDa GP1 band was also accompanied by an ~fourfold increase in LASV titers compared to the titers in cells transfected with control or IFITM3 siRNA (Fig. 3F). No change in NP levels was detected in cells transfected with CH25H siRNA. These results suggested that knocking down CH25H increased the yield of the 42-kDa GP1 species and enhanced LASV replication.

### Aberrantly glycosylated GP1 incorporates into virions.

**FIG 4:**
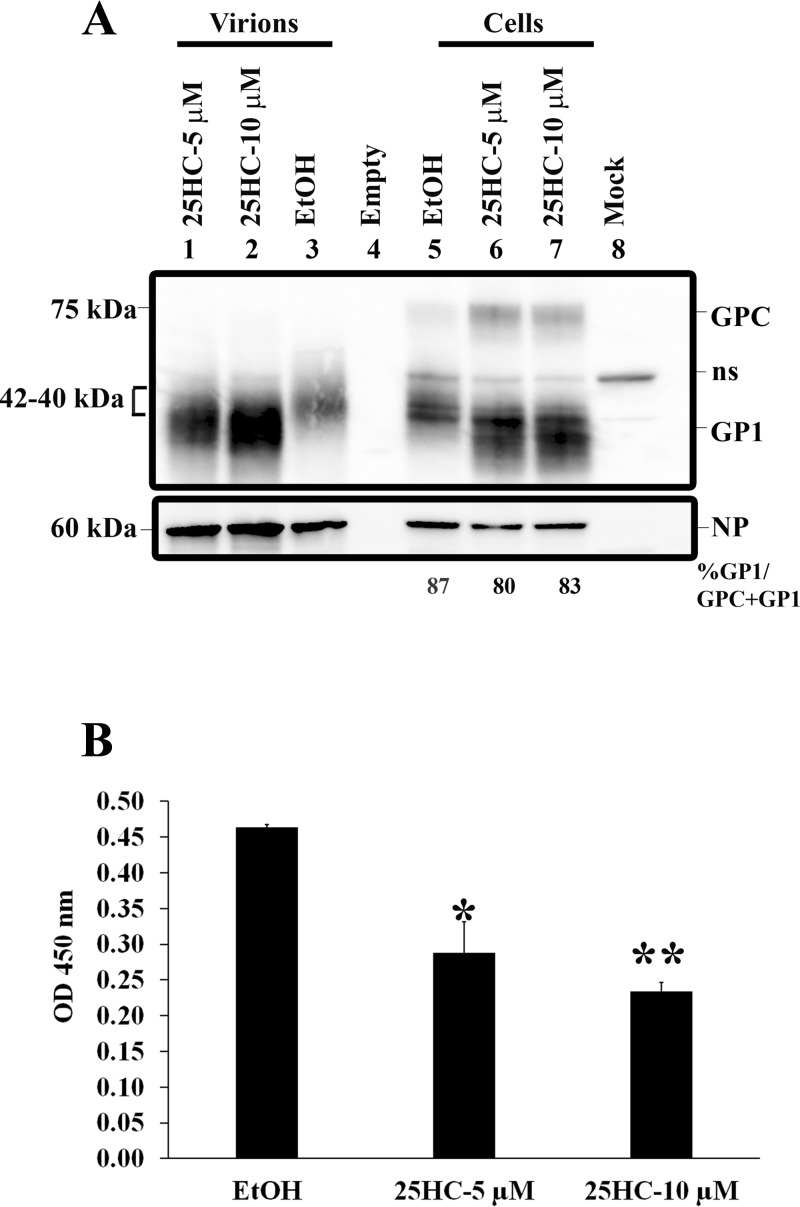
Differentially glycosylated GP1 species are incorporated into LASV particles. (A) Cells treated with ethanol or 25HC were infected with LASV at an MOI of 0.1. Supernatants and cell lysates were collected, and LASV particles were purified from supernatants by ultracentrifugation through a sucrose cushion as described in Materials and Methods. Purified LASV particles were resuspended in 2× sample lysis buffer, and levels of GP1 or NP were analyzed by Western blotting. ns, nonspecific binding. (B) Alpha-DG–Fc fusion proteins were immobilized on 96-well plates and incubated with semipurified LASV preparations obtained from control and 25HC-treated cells. Equal volumes of purified LASV were incubated on alpha-DG, and bound fractions were measured by determining the optical density (OD) at 450 nm. *, *P* < 0.05; **, *P* < 0.005.

A possible cause for reduced LASV infectivity in 25HC-treated cells may be the incorporation of aberrantly glycosylated GP1 into LASV particles. To determine whether virus particles released from 25HC-treated cells contain aberrantly glycosylated GP1, supernatants of control or 25HC-treated cells were sedimented through a 20% sucrose cushion. No uncleaved GPC (~75 kDa) was detected in the pelleted virions (Fig. 4A, left versus right), as previously reported (6). The NP levels were comparable in all samples. Virus released from control cells contained only the typically glycosylated form of GP1, migrating between 40 and 42 kDa. In contrast, in cells treated with 25HC, the majority of GP1 present in the pelleted LASV migrated below 40 kDa, indicating that 25HC treatment resulted in efficient incorporation of aberrantly glycosylated GP1 into LASV particles.

### LASV virions containing aberrantly glycosylated GP1 are defective in alpha-DG binding.

The initial attachment of LASV to the cell surface is dependent on the host glycoprotein alpha-DG. To test whether the aberrantly glycosylated LASV particles produced after 25HC treatment were defective in alpha-DG binding, we performed a binding assay (20). In this assay, alpha-DG–Fc fusion proteins were immobilized and incubated with semipurified LASV preparations obtained from control and 25HC-treated cells. Equal volumes of purified LASV were incubated on alpha-DG, and the bound fractions were measured (Fig. 4B). LASV produced in 25HC-treated cells bound alpha-DG less efficiently than LASV from control cells, despite the overabundance of GP1 present in the particles produced in the presence of 25HC compared to the amount in particles from control cells (Fig. 4A, lane 3 versus lanes 1 and 2). These results suggest that binding of the aberrantly glycosylated form of GP1 to alpha-DG is compromised.

## DISCUSSION

Here, we demonstrate that 25HC has antiviral activity against LASV that is linked to the production of virus particles with reduced infectivity. LASV particles produced in the presence of 25HC contained aberrantly glycosylated GP1, the subunit of the viral glycoprotein responsible for receptor binding. These particles bound poorly to alpha-DG, the cell surface attachment and entry factor for LASV. Thus, 25HC inhibits LASV infection by a novel mechanism that alters the maturation of N-linked glycans, leading to impaired receptor binding.

The correct folding and cleavage of arenavirus GPC are critical for the incorporation of GP1 and GP2 into virions, and inhibition of cleavage results in the release of noninfectious virus particles devoid of surface glycoproteins (6). 25HC reduced LASV infectivity, but not through defective GPC cleavage. Instead, 25HC blocked the maturation of N-linked glycans on the viral glycoprotein subunits, rendering them more sensitive to digestion with Endo-H than the untreated forms; the majority of cell-associated GPC, GP1, and GP2 glycoproteins were sensitive to Endo-H in our experiments, but the sensitivity of GP2 to Endo-H was not affected by 25HC treatment. Although we did not extensively investigate these apparent differences, the more extensive glycosylation of GP1 (GP1 has 7 *N*-glycan sites, versus 4 in GP2) (10) might facilitate the detection of changes in GP1 glycosylation after Endo-H treatment, while changes in GP2 are harder to detect. Since Endo-H only removes immature *N*-glycans that are rich in mannose residues, the addition and/or trimming of *N*-glycans might be crucial for 25HC-induced reduction of LASV infectivity. Indeed, abnormal *N*-glycan maturation due to changing the SKI-/S1P cleavage motif (RSLK) to a furin motif (RRKR) in Junin virus, a New World arenavirus, resulted in reduced viral spread, suggesting that glycan maturation might affect arenavirus infectivity in general (25). The aberrantly glycosylated GP1 was readily incorporated into viral particles, though the ability of these particles to bind alpha-DG was significantly compromised. Although inhibitors of trimming oligomannosyl-rich chains had no effect on the production of infectious LCMV, another arenavirus that uses alpha-DG as a receptor (26), the loss of *N*-glycans in GP1 of LCMV has been associated with reduction in infectivity (11). Mutating 3 of the 4 *N*-glycans of LCMV GP1 produces noninfectious viruslike particles without altering GPC processing, suggesting that GP1’s glycan composition can be critical for the infectivity of other arenaviruses (11). Therefore, we propose that the inhibition of LASV infectivity by 25HC is at least partly due to inefficient binding of the aberrantly glycosylated GP to alpha-DG.

The mechanism by which 25HC induces changes in the GP1 glycans is currently unknown, but we speculate that Golgi-complex and *trans*-Golgi network-associated glycosidases and/or mannosidases involved in GP1 maturation might be affected by 25HC. Since the conversion of high-mannose oligosaccharide to a complex type occurs in the medial to *trans*-Golgi network (27), our data suggest that LASV GP1 is transported to the *cis*-Golgi compartment. This is consistent with our findings that 25HC did not affect LASV GPC cleavage, a process that occurs in the endoplasmic reticulum (ER) and/or *cis*-Golgi compartment. Accordingly, blocking the complex-*N*-glycan maturation of HIV glycoprotein GP120 by inhibiting mannosidase I has been linked to reduced virion infectivity without changes in GP160 cleavage (28). Alternatively, a 25HC-induced alteration of normal transport of GPC or its cleaved products might affect the extent of GP1 glycan maturation, although the release of LASV particles following 25HC treatment appeared unimpaired.

*CH25H* is an interferon-stimulated gene, and its expression is upregulated during infection with hepatitis C and influenza viruses (16, 29). How LASV infection upregulates *CH25H* is unknown, especially since LASV-infected human cells produce undetectable or very low levels of type-I IFN (30, 31). Further investigations are required to understand the mechanism of *CH25H* transcriptional activation by LASV. CH25H knockdown enhanced LASV titers and increased the levels of the ~42-kDa GP1 normally found in the virus. LASV containing the ~42-kDa GP1 had better receptor binding activity than LASV bearing the aberrantly glycosylated GP1 induced by 25HC. This suggests that the enhanced LASV titers seen after CH25H knockdown are probably due to increased LASV infectivity, as CH25H overexpression and 25HC inversely affected both virus titers and glycosylation. However, further experiments are necessary to rule out the possibility that CH25H depletion increases the release of viral particles.

While the exact mechanism by which CH25H inhibits GP1 glycosylation remains to be elucidated, our data demonstrate the importance of CH25H and the unexpected role of GP1 glycosylation in LASV infectivity. Future experiments are warranted to more precisely address the biochemical changes of glycans associated with 25HC treatment and the effect of these changes on LASV’s interactions with its cell surface receptor.

## MATERIALS AND METHODS

### Biosafety.

All work with infectious virus was conducted in a BSL-4 laboratory at the Centers for Disease Control and Prevention (CDC, Atlanta, GA), following the guidelines of CDC standard operating procedures.

### Cells, virus, plasmids, reagents, and antibodies.

Vero-E6 cells were maintained in Dulbecco’s modified Eagle’s medium (DMEM; Life Technologies, Inc., Grand Island, NY, USA) supplemented with 10% (vol/vol) fetal calf serum (FCS, HyClone; Thermo Scientific, Waltham, MA, USA) and penicillin-streptomycin (Life Technologies, Inc.). In some samples, sterol-depleted medium (lipoprotein-deficient serum; Sigma-Aldrich, St. Louis, MO, USA) was used instead of medium with FCS. Huh7 cells were propagated in DMEM with 10% (vol/vol) FCS and 1× nonessential amino acids (Life Technologies, Inc.). 25HC was from Sigma-Aldrich. LASV (Josiah strain) was from the CDC Viral Special Pathogens Branch reference collection; all work with LASV was done under BSL-4 conditions. The plasmid encoding LASV-GPC has been previously described (21, 32). Plasmids encoding Myc-tagged CH25H and Myc-tagged IFITM3 were purchased from Origene (Rockville, MD, USA). The following antibodies were used in this study: mouse monoclonal antibody against LASV NP (703079-Lassa Josiah HMAF), mouse monoclonal antibody against LASV GP1 (52-74-7), monoclonal antibody against LASV GP2 (81001-52-2085-0006-BG12-alpha-LASVG2), and a mix of 5 monoclonal LASV antibodies for immunofluorescence (SPR628 anti-LASV 5-MAb mixture). The anti-Myc antibody was from Origene, and the mouse monoclonal antiactin antibody was from Sigma-Aldrich (St. Louis, MO, USA).

### Transfection and infection.

To determine the effects of 25HC on GP1 glycosylation in LASV GPC-transfected cells, Huh7 cells were plated at 2.5 × 10^5^ per well in 12-well plates. The following day, cells were transiently transfected with 2 µg of empty expression vector (pCAGGS) or with a plasmid expressing LASV-GPC at the indicated concentrations, using TransIT-LT1 according to the manufacturer’s instructions (Mirus, Madison, WI, USA). After 24 h, cells were treated with 25HC; cells were harvested 48 h posttransfection. To determine the effects of CH25H and IFITM3 overexpression on LASV GPC, Huh7 cells were transfected with either empty vector (pCMV6) or plasmid expressing Myc-tagged CH25H or Myc-tagged IFITM3 using TransIT-LT1 (Mirus). After 24 h, cells were washed twice with DMEM and then retransfected with plasmid expressing LASV GPC as described above.

For LASV infection, Huh7 cells were plated at 5 × 10^5^ cells per well in 12-well plates. The next day, cells were treated with ethanol or 25HC in serum-free medium for 1 h. 25HC treatment was done in serum-free medium to enhance the effect of the drug by avoiding potential binding of 25HC to serum lipids and proteins. Cells were then infected with LASV at the indicated MOIs for 1 h. The virus inoculum was removed, and cells were washed with serum-free medium. Fresh medium containing 2% FCS and with or without 25HC was then added. To determine the effects of CH25H or IFITM3 on LASV infection, cells were transfected with either empty vector (pCMV6) or plasmid expressing Myc-tagged CH25H or Myc-tagged IFITM3 using TransIT-LT1 (Mirus) as described above. After 24 h, transfected cells were washed twice with DMEM and then infected with LASV at an MOI of 0.5.

Huh7 cells (2.5 × 10^5^ per well) were reverse transfected and then retransfected 48 h later using DharmaFect 1 (Thermo Fisher Scientific) and 100 nmol siRNA against CH25H or IFITM3 or control siRNA (ON-Target plus SMARTpool siRNA; Thermo Fisher Scientific). Seventy-two hours after the initial transfection, cells were infected with LASV at an MOI of 1 under serum-free conditions. After 1 h of virus adsorption, cells were washed 4 times with serum-free medium and then replenished with DMEM containing 5% lipoprotein-deficient serum.

### TCID_50_ determination.

Supernatants from LASV-infected cells were harvested at the indicated time points, and virus titrations were performed in Vero-E6 cells. Three days postinfection, the cells were fixed, permeabilized, and stained to visualize viral proteins. Endpoint viral titers were determined, and TCID_50_s were calculated as described previously (21). The results represent the mean titers and standard deviations calculated from the results of 3 independent experiments.

### Quantitative RT-PCR.

Huh7 cells were infected with LASV for the indicated times, and then RNA was isolated from cells or from supernatants of infected cells using the MagMAX-96 total RNA isolation kit (Thermo Fisher Scientific). One-step qRT-PCR was performed using an Applied Biosystems 7500 real-time PCR system and primers specific for CH25H or IFITM3 from single-tube TaqMan assays (Thermo Fisher Scientific). The levels of target genes were normalized to the GAPDH levels using control primer-probe sets (Thermo Fisher Scientific). The fold modulations in gene expression of CH25H and IFITM3 mRNA were analyzed using the cycle threshold (2^−ΔΔ*CT*^) method, comparing the levels of target mRNA in LASV-infected cells with the levels in mock-infected cells. The results shown are the means of CH25H mRNA expression in 3 independent experiments. To determine viral RNA copy numbers, RNA was extracted from the supernatants of infected cells. Absolute quantification of viral RNA copy numbers was done by measuring LASV S segment copy numbers using a standard curve with known viral titers serially diluted fivefold. One-step qRT-PCR was conducted using LASV S segment-specific forward (5′-AATCAGTTCGGGACCATGC-3′) and reverse (5′-GTGTTGGGATACTTTGCTGTG-3′) primers and a probe oligonucleotide (5′-/56-FAM/AGTCAACCT/ZEN/GCCCCTGTTTTGTCA/Iowa Black FQ/-3′ [6-FAM is 6-carboxyfluorescein]) from Integrated DNA Technologies, Inc. (Coralville, IA, USA).

### Western blotting.

At the indicated times after transfection or infection, cell lysates were harvested by adding lysis buffer containing 50 mM NaCl, 5 mM EDTA, 1% NP-40, 0.1% SDS, and 0.5% sodium deoxycholate supplemented with a protease inhibitor cocktail (Sigma-Aldrich). Lysates from infected cells were gamma irradiated at 2 × 10^6^ rad using a high-energy ^60^Co source. Proteins were electrophoretically separated on 4-to-12% NuPAGE gels (Invitrogen) and transferred to nitrocellulose membranes. The membranes were blocked for 1 h with buffer containing Tris-buffered saline, 0.1% Tween 20, and 5% nonfat dry milk and then probed overnight at 4°C with primary antibodies. The membranes were developed using horseradish peroxidase-conjugated secondary antibodies and enhanced chemiluminescence. After the detection of primary antibodies, the membranes were stripped and reprobed with antiactin antibody as a loading control. The results shown are representative of 3 independent experiments.

### Virion purification.

Supernatants from ethanol- or 25HC-treated and LASV-infected cells were clarified by centrifuging at 1,500 × *g* for 30 min. Clarified supernatants were subjected to ultracentrifugation (100,000 × *g* for 90 min at 4°C) through a 20% sucrose cushion to collect LASV virions. Virions were suspended in 2× Western lysis buffer, gamma irradiated at 5 × 10^6^ rad using a high-energy ^60^Co source, and analyzed by Western blotting to detect GP1 and NP. Alternatively, virions were suspended in phosphate-buffered saline (PBS) and used for alpha-DG fusion protein binding enzyme-linked immunosorbent assay (ELISA) under BSL-4 conditions.

### Lassa virions and alpha-DG–Fc fusion protein binding ELISA.

A plasmid expressing a fusion protein consisting of the signal sequence from CD5, the ectodomain of alpha-DG (amino acids 30 to 430), and the Fc domain of human IgG1 was generated by cloning the alpha-DG sequence amplified from RNA extracted from HEK-293 cells into plasmid pSyngp120IgG (obtained through the NIH AIDS Research and Reference Reagent Program, Division of AIDS, NIAID, NIH, from Eun-Chung Park and Brian Seed). The expression and purification of the DG3.5-Fc fusion protein were performed by GenScript (Piscataway, NJ). Briefly, the expression plasmid was transiently transfected into a suspension of HEK-293 cells. The fusion protein was captured from the culture supernatant on a HiTrap protein A HP column according to the manufacturer’s instructions (GE Healthcare). After washing with 20 mM sodium phosphate, the protein was eluted with 3 M MgCl_2_ before buffer exchange to PBS. Binding of LASV to the alpha-DG–Fc fusion protein was done using a protocol described previously, with the following modifications (20). Briefly, microtiter plates were coated with 2 μg/ml alpha-Dg–Fc fusion protein overnight at 4°C. The coated plates were blocked for nonspecific binding using 1% bovine serum albumin (BSA)–PBS. Equal volumes of partially purified LASV were applied for 24 h at 4°C. For detection of bound virus, a mixture of MAbs against GP1 (50 μg/ml) and GP2 (1:10 dilution) was used in 1% BSA–PBS. Bound primary antibodies were detected with peroxidase-conjugated secondary antibody against tetramethylbenzidine (TMB) substrate. The optical density at 450 nm was determined using a Synergy H1MD plate reader (BioTek, Winooski, VT, USA).

### Endo-H and PNGase F treatment.

In order to investigate the modifications of LASV glycoproteins, cell lysates were digested with Endo-H or PNGase F (NEB, Ipswich, MA, USA) according to the manufacturer’s instructions. The digested proteins were resolved by SDS-PAGE under reducing conditions and were analyzed by Western blotting.

Error bars in all graphs represent standard deviations of the means, with *P* values from Student’s *t* tests indicated (*, *P* < 0.05; **, *P* < 0.005).

## SUPPLEMENTAL MATERIAL

Figure S1  25HC does not affect LASV cell entry. Huh7 cells were infected with LASV (MOI 1.0) in the presence of indicated concentrations of 25HC, BIBX 1382, or vehicle (ethanol or dimethyl sulfoxide [DMSO]). After 1 h, infected cells were washed with serum-free medium; fresh medium with or without 25HC was then added, and cells were incubated at 37°C for 1 h. After 1 h, the virus inoculum was removed, the cells were washed, and fresh medium containing either ethanol or 25HC was added. After 2 h of incubation, the levels of S segment RNA were determined by qRT-PCR and normalized to the level of GAPDH mRNA. Values are expressed as % normalized RNA, with error bars indicating standard deviations calculated from the results of 3 independent experiments. Download Figure S1, TIF file, 0.4 MB

Figure S2  Twenty-four hours of treatment with 25HC does not affect LASV GP1 glycosylation. Huh7 cells transfected with a plasmid expressing LASV GPC were treated with 25HC for 24 h. The levels of GP1 and actin expression in cell lysates were then analyzed by Western blotting. Download Figure S2, TIF file, 1.4 MB

## References

[1] Ogbu O, Ajuluchukwu E, Uneke CJ 2007 Lassa fever in West African sub-region: an overview. J Vector Borne Dis 44:1–11. 17378212

[2] Richmond JK, Baglole DJ 2003 Lassa fever: epidemiology, clinical features, and social consequences. BMJ 327:1271–1275. doi:10.1136/bmj.327.7426.1271. 14644972 PMC286250

[3] Werner GT 1977 What is Lassa fever? Med Klin 72:1786 (In German.). 916963

[4] McCormick JB, King IJ, Webb PA, Scribner CL, Craven RB, Johnson KM, Elliott LH, Belmont-Williams R 1986 Lassa fever. Effective therapy with ribavirin. N Engl J Med 314:20–26. doi:10.1056/NEJM198601023140104. 3940312

[5] CDC. 2015 Lassa fever confirmed in death of U.S. traveler returning from Liberia. CDC, Atlanta, GA http://www.cdc.gov/media/releases/2015/p0525-lassa.html.

[6] Lenz O, ter Meulen J, Klenk HD, Seidah NG, Garten W 2001 The Lassa virus glycoprotein precursor GP-C is proteolytically processed by subtilase SKI-1/S1P. Proc Natl Acad Sci U S A 98:12701–12705. doi:10.1073/pnas.221447598. 11606739 PMC60117

[7] Burri DJ, Pasqual G, Rochat C, Seidah NG, Pasquato A, Kunz S 2012 Molecular characterization of the processing of arenavirus envelope glycoprotein precursors by subtilisin kexin isozyme-1/site-1 protease. J Virol 86:4935–4946. doi:10.1128/JVI.00024-12. 22357276 PMC3347368

[8] Burri DJ, da Palma JR, Kunz S, Pasquato A 2012 Envelope glycoprotein of arenaviruses. Viruses 4:2162–2181. doi:10.3390/v4102162. 23202458 PMC3497046

[9] Bowen MD, Rollin PE, Ksiazek TG, Hustad HL, Bausch DG, Demby AH, Bajani MD, Peters CJ, Nichol ST 2000 Genetic diversity among Lassa virus strains. J Virol 74:6992–7004. doi:10.1128/JVI.74.15.6992-7004.2000. 10888638 PMC112216

[10] Eichler R, Lenz O, Garten W, Strecker T 2006 The role of single N-glycans in proteolytic processing and cell surface transport of the Lassa virus glycoprotein GP-C. Virol J 3:41. doi:10.1186/1743-422X-3-41. 16737539 PMC1524727

[11] Bonhomme CJ, Capul AA, Lauron EJ, Bederka LH, Knopp KA, Buchmeier MJ 2011 Glycosylation modulates arenavirus glycoprotein expression and function. Virology 409:223–233. doi:10.1016/j.virol.2010.10.011. 21056893 PMC3053032

[12] Rojek JM, Pasqual G, Sanchez AB, Nguyen NT, de la Torre JC, Kunz S 2010 Targeting the proteolytic processing of the viral glycoprotein precursor is a promising novel antiviral strategy against arenaviruses. J Virol 84:573–584. doi:10.1128/JVI.01697-09. 19846507 PMC2798452

[13] Holmes RS, Vandeberg JL, Cox LA 2011 Genomics and proteomics of vertebrate cholesterol ester lipase (LIPA) and cholesterol 25-hydroxylase (CH25H). 3 Biotech 1:99–109. doi:10.1007/s13205-011-0013-9. PMC3324826 22582164

[14] Liu SY, Aliyari R, Chikere K, Li G, Marsden MD, Smith JK, Pernet O, Guo H, Nusbaum R, Zack JA, Freiberg AN, Su L, Lee B, Cheng G 2013 Interferon-inducible cholesterol-25-hydroxylase broadly inhibits viral entry by production of 25-hydroxycholesterol. Immunity 38:92–105. doi:10.1016/j.immuni.2012.11.005. 23273844 PMC3698975

[15] Blanc M, Hsieh WY, Robertson KA, Kropp KA, Forster T, Shui G, Lacaze P, Watterson S, Griffiths SJ, Spann NJ, Meljon A, Talbot S, Krishnan K, Covey DF, Wenk MR, Craigon M, Ruzsics Z, Haas J, Angulo A, Griffiths WJ, Glass CK, Wang Y, Ghazal P 2013 The transcription factor STAT-1 couples macrophage synthesis of 25-hydroxycholesterol to the interferon antiviral response. Immunity 38:106–118. doi:10.1016/j.immuni.2012.11.004. 23273843 PMC3556782

[16] Xiang Y, Tang JJ, Tao W, Cao X, Song BL, Zhong J 2015 Identification of cholesterol 25-hydroxylase as a novel host restriction factor and a part of the primary innate immune responses against hepatitis C virus infection. J Virol 89:6805–6816. doi:10.1128/JVI.00587-15. 25903345 PMC4468479

[17] Anggakusuma, Romero-Brey I, Berger C, Colpitts CC, Boldanova T, Engelmann M, Todt D, Perin PM, Behrendt P, Vondran FW, Xu S, Goffinet C, Schang LM, Heim MH, Bartenschlager R, Pietschmann T, Steinmann E 2015 Interferon-inducible cholesterol-25-hydroxylase restricts hepatitis C virus replication through blockage of membranous web formation. Hepatology 62:702–714. doi:10.1002/hep.27913. 25999047

[18] Civra A, Cagno V, Donalisio M, Biasi F, Leonarduzzi G, Poli G, Lembo D 2014 Inhibition of pathogenic non-enveloped viruses by 25-hydroxycholesterol and 27-hydroxycholesterol. Sci Rep 4:7487. doi:10.1038/srep07487. 25501851 PMC4265783

[19] Cao W, Henry MD, Borrow P, Yamada H, Elder JH, Ravkov EV, Nichol ST, Compans RW, Campbell KP, Oldstone MB 1998 Identification of alpha-dystroglycan as a receptor for lymphocytic choriomeningitis virus and Lassa fever virus. Science 282:2079–2081. doi:10.1126/science.282.5396.2079. 9851928

[20] Kunz S, Rojek JM, Perez M, Spiropoulou CF, Oldstone MB 2005 Characterization of the interaction of Lassa fever virus with its cellular receptor alpha-dystroglycan. J Virol 79:5979–5987. doi:10.1128/JVI.79.10.5979-5987.2005. 15857984 PMC1091707

[21] Mohr EL, McMullan LK, Lo MK, Spengler JR, Bergeron É, Albariño CG, Shrivastava-Ranjan P, Chiang CF, Nichol ST, Spiropoulou CF, Flint M 2015 Inhibitors of cellular kinases with broad-spectrum antiviral activity for hemorrhagic fever viruses. Antiviral Res 120:40–47. doi:10.1016/j.antiviral.2015.05.003. 25986249

[22] Roberts DL, Weix DJ, Dahms NM, Kim JJ 1998 Molecular basis of lysosomal enzyme recognition: three-dimensional structure of the cation-dependent mannose 6-phosphate receptor. Cell 93:639–648. doi:10.1016/S0092-8674(00)81192-7. 9604938

[23] Desai TM, Marin M, Chin CR, Savidis G, Brass AL, Melikyan GB 2014 IFITM3 restricts influenza A virus entry by blocking the formation of fusion pores following virus-endosome hemifusion. PLoS Pathog 10:e1004048. doi:10.1371/journal.ppat.1004048. 24699674 PMC3974867

[24] Amini-Bavil-Olyaee S, Choi YJ, Lee JH, Shi M, Huang IC, Farzan M, Jung JU 2013 The antiviral effector IFITM3 disrupts intracellular cholesterol homeostasis to block viral entry. Cell Host Microbe 13:452–464. doi:10.1016/j.chom.2013.03.006. 23601107 PMC3646482

[25] Albariño CG, Bergeron E, Erickson BR, Khristova ML, Rollin PE, Nichol ST 2009 Efficient reverse genetics generation of infectious Junin viruses differing in glycoprotein processing. J Virol 83:5606–5614. doi:10.1128/JVI.00276-09. 19321606 PMC2681955

[26] Wright KE, Spiro RC, Burns JW, Buchmeier MJ 1990 Post-translational processing of the glycoproteins of lymphocytic choriomeningitis virus. Virology 177:175–183. doi:10.1016/0042-6822(90)90471-3. 2141203 PMC7130728

[27] Kornfeld R, Kornfeld S 1985 Assembly of asparagine-linked oligosaccharides. Annu Rev Biochem 54:631–664. doi:10.1146/annurev.bi.54.070185.003215. 3896128

[28] Pal R, Hoke GM, Sarngadharan MG 1989 Role of oligosaccharides in the processing and maturation of envelope glycoproteins of human immunodeficiency virus type 1. Proc Natl Acad Sci U S A 86:3384–3388. doi:10.1073/pnas.86.9.3384. 2541446 PMC287137

[29] Gold ES, Diercks AH, Podolsky I, Podyminogin RL, Askovich PS, Treuting PM, Aderem A 2014 25-Hydroxycholesterol acts as an amplifier of inflammatory signaling. Proc Natl Acad Sci U S A 111:10666–10671. doi:10.1073/pnas.1404271111. 24994901 PMC4115544

[30] Asper M, Sternsdorf T, Hass M, Drosten C, Rhode A, Schmitz H, Günther S 2004 Inhibition of different Lassa virus strains by alpha and gamma interferons and comparison with a less pathogenic arenavirus. J Virol 78:3162–3169. doi:10.1128/JVI.78.6.3162-3169.2004. 14990737 PMC353741

[31] Müller S, Geffers R, Günther S 2007 Analysis of gene expression in Lassa virus-infected HuH-7 cells. J Gen Virol 88:1568–1575. doi:10.1099/vir.0.82529-0. 17412988

[32] Albariño CG, Bird BH, Chakrabarti AK, Dodd KA, White DM, Bergeron E, Shrivastava-Ranjan P, Nichol ST 2011 Reverse genetics generation of chimeric infectious Junin/Lassa virus is dependent on interaction of homologous glycoprotein stable signal peptide and G2 cytoplasmic domains. J Virol 85:112–122. doi:10.1128/JVI.01837-10. 20980515 PMC3014187

