# Supplementary figures and images for: 25-Hydroxycholesterol Inhibition of Lassa Virus Infection through Aberrant GP1 Glycosylation
Source: mBio. 2016 Dec 20;7(6):e01808-16. doi: 10.1128/mBio.01808-16 (PMC5181775; doi:10.1128/mBio.01808-16)

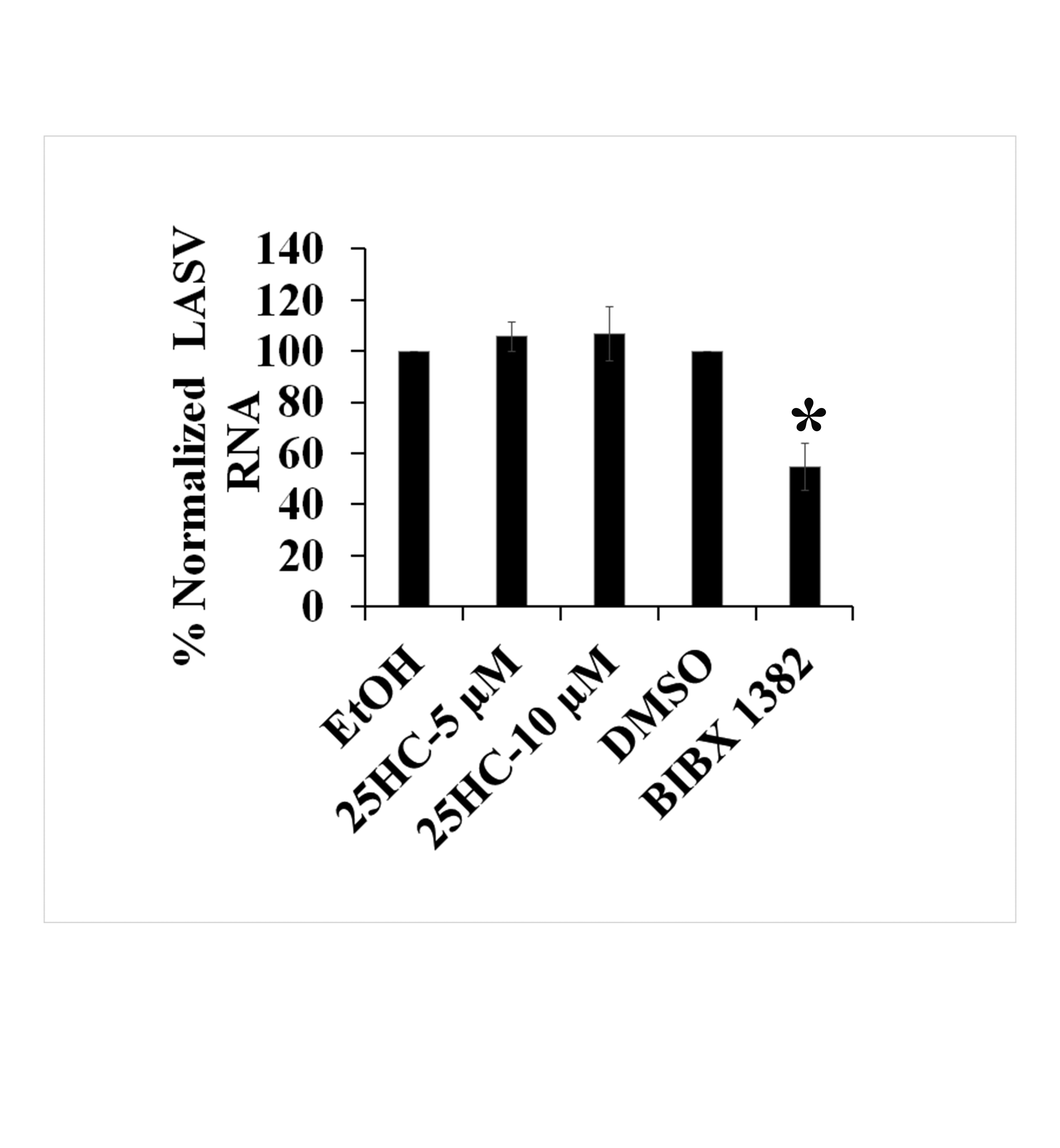

Supplement: Figure S1 — 25HC does not affect LASV cell entry. Huh7 cells were infected with LASV (MOI 1.0) in the presence of indicated concentrations of 25HC, BIBX 1382, or vehicle (ethanol or dimethyl sulfoxide [DMSO]). After 1 h, infected cells were washed with serum-free medium; fresh medium with or without 25HC was then added, and cells were incubated at 37°C for 1 h. After 1 h, the virus inoculum was removed, the cells were washed, and fresh medium containing either ethanol or 25HC was added. After 2 h of incubation, the levels of S segment RNA were determined by qRT-PCR and normalized to the level of GAPDH mRNA. Values are expressed as % normalized RNA, with error bars indicating standard deviations calculated from the results of 3 independent experiments. Download [file mbo006163117sf1.tif]

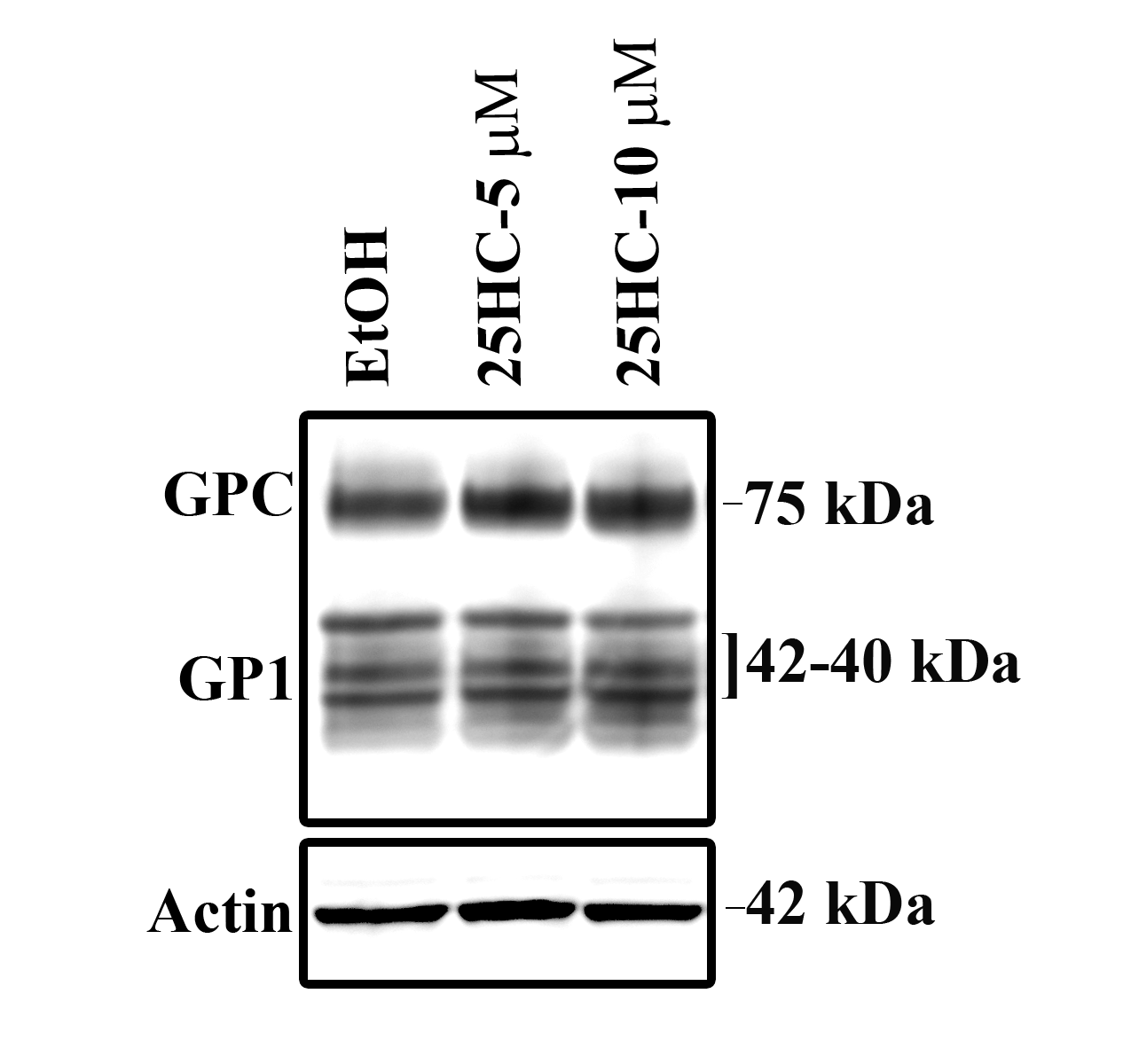

Supplement: Figure S2 — Twenty-four hours of treatment with 25HC does not affect LASV GP1 glycosylation. Huh7 cells transfected with a plasmid expressing LASV GPC were treated with 25HC for 24 h. The levels of GP1 and actin expression in cell lysates were then analyzed by Western blotting. Download [file mbo006163117sf2.tif]
